# Untangling the evolutionary roots of lung cancer

**DOI:** 10.1038/s41467-019-10879-6

**Published:** 2019-07-05

**Authors:** Siddhartha Devarakonda, Ramaswamy Govindan

**Affiliations:** 1 Division of Oncology, Washington University School of Medicine in St. Louis, St. Louis, MO 63110 United States; 2 Siteman Cancer Center, St. Louis, MO 63110 United States

**Keywords:** Cancer genomics, Lung cancer, Cancer prevention

## Abstract

The genomic and host factors that drive the progression of pre-invasive lesions in non-small cell lung cancer are poorly understood. Studying these factors can advance our knowledge of lung cancer biology, aid in the development of better screening strategies and improve patient outcomes.

## Early detection of lung cancer

Lung cancer is one of the leading causes of cancer related mortality^1^. Nearly 85% of patients with lung cancer are diagnosed with non-small cell lung cancer (NSCLC), of which lung adenocarcinoma (LUAD) and squamous cell carcinoma (SQCC) are the commonly diagnosed subtypes. A majority of patients with NSCLC are diagnosed with metastatic disease at presentation. Screening high-risk patients (defined by the National Lung Screening Trial as current smokers or those who quit smoking within 15 years, aged 55–74 years, with a 30 pack-year smoking history) for lung cancer through annual low-dose CT (LDCT) scanning is associated with a reduction in lung cancer related mortality^2,3^. Nearly two-thirds of the cancers identified in these screening studies were stage I cancers, which are potentially curable through resection. However, LDCT screening is associated with a high false positive rate, suggesting the need for combining imaging with other strategies, such as molecular testing, to improve screening efficiency, decrease costs, and minimize procedural risks associated with confirmatory biopsies. While pre-invasive lesions in NSCLC have been well described, not much is known about their biology and factors that drive their progression to cancer. This knowledge can help develop screening strategies aimed at identifying pre-invasive lesions at high risk for progression and offer an opportunity for early intervention.

## Pre-invasive lung cancer lesions

The airway epithelium is heterogeneous and consists of different cell types. The final histology of a lung cancer is probably determined by both the cell from which cancer arises and specific molecular alterations that drive transformation^4^. Pre-invasive lesions have been described for both LUADs and SQCCs. Atypical adenomatous hyperplasia (AAH) and Adenocarcinoma-in-situ (AIS) are recognized as pre-invasive lesions for LUAD^5^. AAHs have been incidentally identified in the lung parenchyma adjacent to 5–23% of resected LUADs^5^. These lesions represent a localized proliferation of atypical appearing type 2 alveolar and club cells and measure <0.5 cm. AIS is a term used to describe neoplastic cells growing along pre-existing alveolar structures (lepidic pattern) and measure <3 cm. Unlike AIS, lesions that demonstrate a focus of invasion measuring <0.5 cm are described as minimally invasive adenocarcinomas (MIA). AAH and AIS appear as ground glass opacities on CT imaging, while MIAs additionally demonstrate a solid component. These lesions likely constitute a continuum along which pre-invasive lesions progress to LUAD. Similarly, low and high-grade squamous dysplasia and carcinoma-in-situ have been described as pre-invasive lesions for SQCC.

Surgical removal of pre-invasive lesions is associated with 100% 5-year survival. However, identifying these lesions is challenging and interventions aimed at their removal can be fairly invasive. Furthermore, data from longitudinal studies indicate that as many as 54% of pre-invasive lesions can regress without progressing to cancer, while 0.8–39% of lesions can progress to cancer depending on the grade of dysplasia^6,7^. It is therefore, crucial to gain a thorough understanding of the molecular features predicting progression for developing effective therapeutic strategies and avoiding over-treatment.

The ability of a pre-invasive lesion to successfully establish invasive disease is likely determined by a variety of tumor intrinsic and host factors. While some of the processes that drive progression are shared between LUAD and SQCC, other processes tend to be sub-type specific. For instance, SQCCs appear to be characterized by early acquisition of 3q gain and loss of expression of NKX2-1, a lineage survival oncogene for LUAD, while LUADs select for alterations that activate RTK/RAS signaling^8^. Large scale next-generation sequencing studies, have elegantly demonstrated the ability of whole-exome and multi-regional sequencing to display the clonal architecture of a cancer and deduce the sequence in which cancer cells acquire genomic alterations^9,10^. Together, data from these and other similar studies have highlighted several common themes in the evolution of NSCLC^10–12^. For instance, alterations in genes such as *TP53, KRAS, EGFR* and *BRAF* occur early in the course of tumor evolution. Nearly, three-quarters of lung cancers undergo genome duplication, which probably allows them to withstand and propagate additional genomic instability. NSCLCs often acquire mutations in other genes crucial for malignant transformation, such as those regulating chromatin remodeling, histone methylation, and DNA damage response, later in the course of their evolution. NSCLCs also demonstrate heterogeneity in the mutational processes that drive tumor evolution. Owing to the mutagenic effect of cigarette smoke, clonal alterations in smoking related lung cancer are typically enriched for C > A transversions, while sub-clonal mutations show C > T transitions driven by APOBEC (cytidine deaminases) enzymes and/or aging related spontaneous deamination. Limited data of such nature exists for pre-invasive lesions.

## Genomic features of pre-invasive lesions and LUADs

Now, Hu and colleagues report on results from multiregional, whole-exome sequencing of pre-invasive lesions and LUADs^13^. Results from this study demonstrate that mutation burden increases as lesions in the airway progress from AAH to invasive disease. This is reflected both in the increase of clonal and sub-clonal mutations. As expected, this increase appeared more notable in smokers compared to never-smokers. Mutagenic processes driven by smoking, DNA repair defects, aging associated C > T deamination, and APOBEC, appeared to be operational in pre-invasive lesions. Enrichment for APOBEC driven mutagenesis was observed as lesions progressed from AAH to LUAD, suggesting that these enzymes play a key role in lung cancer, apart from driving sub-clonal diversification once the disease is established. Additionally, increasing copy number instability and allelic imbalance were also features of malignant progression. These results are in agreement with observations made in pre-invasive lesions that progress to SQCC, with progressive accumulation of mutations and chromosomal instability playing an important role in the establishment of invasive disease^8^.

Chromosomal instability, the progressive accumulation of copy number changes, and allelic imbalance confer several growth advantages to cancer cells. In SQCC, upregulation of genes driving chromosomal instability was found in progressive dysplastic lesions^8^. Large scale genomic analyses utilizing multiple cancer samples suggest that activating oncogenic mutations can themselves drive allelic imbalance by favoring loss of wild type alleles or gain of mutant alleles^14^. Alternately, some cancers evolve and acquire oncogenic mutations and copy number changes separately, with cells co-opting these alterations to produce a mutant allele dosage that confers a survival advantage.

## Evading host immunity

Allelic imbalance plays an important role in tumor immune evasion. Data from the TRACERx study demonstrates that loss of heterozygosity (LOH) of either maternal or paternal HLA alleles is seen in nearly 40% of NSCLCs^15^. HLA LOH is enriched in metastatic lesions and the allele that is lost, is often the allele that is predicted to bind neo-epitopes that are generated from sub-clonal mutations. Considering that HLA LOH was associated with features reported to be characteristic of progressive malignant transformation by Hu and colleagues, such as a high sub-clonal mutation burden and APOBEC mutagenesis, it is likely that processes such as chromosomal instability and allelic imbalance play a key role in aiding the progression of pre-invasive lesions and sub-clonal diversification. Finally, recent observations also suggest that chromosomal instability plays an important role in metastatic dissemination^16^. The importance of acquiring alterations that enable immune evasion in airway pre-invasive lesions was also demonstrated in a recent analysis published by Beane and colleagues^17^. RNA-sequencing of pre-malignant airway lesions and brushings of normal appearing bronchial mucosa in patients undergoing lung cancer screening, showed progressive/persistent pre-malignant dysplastic lesions to be associated with a “Proliferative subtype” signature that is characterized by decreased expression of interferon signaling and antigen presentation pathway genes and depletion of adaptive and innate immune cells, when compared to regressive lesions. Notably, the presence of the proliferative gene signature in normal appearing bronchial brushings was able to predict for presence of proliferative pre-malignant lesions elsewhere in the airway.

Overall, these data suggest that pre-cancerous lesions in the airway undergo a series of complex changes with common patterns including a progressive accumulation of mutations driven by deregulation of APOBECs, copy number changes, immune evasion and selection of alterations that drive RAS/RTK pathway activation, which is a hallmark feature of most LUADs. It is likely that some of these processes are inter-linked. For instance, apart from driving mutagenesis, APOBEC enzymes are capable of promoting chromosomal instability by inducing DNA replication stress^18^. Furthermore, as shown by Hu and colleagues, the evolutionary trajectory of LUAD appears to differ between smokers and never-smokers^13^.

## Future directions in identifying pre-invasive lesions

These findings open several possibilities for clinical application. For instance, therapeutic strategies that target APOBEC mutagenesis are now being actively studied and it is likely that these strategies could play an important role in chemo-prevention. Identifying specific clonal alterations that are acquired early during malignant transformation and are critical for transformation, can aid screening—potentially through the integration of imaging with diagnostic approaches such as cell free DNA testing. Results from the large, multicenter, observational, Circulating Cell-free Genome Atlas (CCGA) study is likely to shed more light on the utility of this approach in aiding early cancer detection (NCT02889978). Identifying key clonal alterations will also provide us the ability to test approaches that can potentially harness host immunity for targeting pre-invasive disease. Integrating clinical and multi-dimensional “—omic” data can help develop scoring systems that are capable of identifying pre-invasive lesions most likely to progress to malignancy, to safely direct invasive therapies^8^. Finally, a systematic study of oncogenic pathways that are activated by allelic imbalance is likely to aid in the discovery of therapeutic targets that are active in pre-invasive and established invasive disease^19^.
